# Scapegoating in Healthcare: A Primitive Response to Tragedy

**DOI:** 10.1177/00207640251398277

**Published:** 2025-12-26

**Authors:** Rachel Gibbons

**Affiliations:** 1 Independent Clinical Researcher, Brighton, UK; 2 Middlesex University, London, UK; 3 Institute of Psychoanalysis, Brighton, UK

**Keywords:** scapegoating, healthcare accountability, tragedy, blame, just culture

## Abstract

**Background:**

In recent years, a pattern has emerged in healthcare systems where clinicians and organisations are publicly blamed and punished following tragic events such as suicides or homicides. These responses often reflect more than institutional accountability, they reveal unconscious cultural mechanisms of scapegoating rooted in collective anxiety, grief, and the intolerability of uncertainty.

**Aims:**

This paper explores scapegoating in healthcare not merely as an ethical or organisational failure, but as a deep-seated psychological and sociocultural process. It aims to illuminate how blame is mobilised as a primitive defence against psychic pain and systemic dysfunction, and to consider the implications for clinical practice, regulation, and public trust.

**Methodological Approach:**

This article employs an interpretive narrative approach, synthesising psychoanalytic and sociological theory to illuminate scapegoating in healthcare. It draws on the work of Freud, Bion, Menzies-Lyth, Douglas, and Girard, and integrates contemporary case material from international healthcare refined through peer discussion to illustrate and develop the theoretical analysis.

**Key Arguments:**

Scapegoating functions as a symbolic ritual of emotional discharge that obstructs learning and distorts justice. Organisational investigations and media narratives often collapse systemic complexity into linear blame, serving retributive rather than restorative purposes. Those targeted are frequently among the most vulnerable, socially, professionally, or structurally. The paper argues that only by recognising and resisting these unconscious group dynamics can healthcare systems foster genuine accountability, psychological safety, and real learning.

And Aaron shall lay both his hands upon the head of the live goat, and confess over him all . . . and all their transgressions in all their sins, putting them upon the head of the goat, and shall send him away . . . into the wilderness.

(The Holy Bible, 1769, Leviticus 16:21)

## Implications

To protect both staff and patients, post-incident processes must prioritise complexity, reparation, and systemic analysis over punishment. The paper calls for reforms in clinical governance, legal accountability, and media engagement, and proposes a framework for identifying scapegoating in real time. Without such changes, the mental health workforce remains at risk of symbolic sacrifice under the guise of accountability.

## Introduction

Scapegoating in healthcare is not new, but the urgency to confront it has grown in an era of high profile tragedies, chronic underfunding, rising demand, and relentless scrutiny. When an unpredictable tragedy occurs an almost reflexive cultural response is triggered: someone must be to blame. Reinforced by the media, politicians, and regulators, this impulse seeks emotional resolution through the symbolic punishment of an individual. But in doing so deeper truths are obscured, about systemic dysfunction, the limits of clinical care, the nature of mortality, and the unsettling unpredictability of human nature.

Healthcare operates under impossible expectations: to predict the unpredictable, control the uncontrollable, and prevent all harm (Gibbons, 2025b; Reason, 2000). When it fails, as it inevitably must, the psychic distress demands containment and scapegoating often becomes the mechanism by which this occurs (Freud, 1915; Girard, 1986). A clinician is isolated, scrutinised, and blamed. This offers the seductive illusion of moral clarity, but at the expense of truth, learning, and justice (Williams, 2018).

This paper examines scapegoating not simply as a failure of organisational ethics or leadership, but as a deeper cultural and psychological phenomenon. It considers how institutions unconsciously reproduce ancient sacrificial logic under the guise of “accountability.” The symbolic roots of scapegoating are described, from biblical ritual to modern legal and media practices, before exploring how these dynamics play out in contemporary health services. It is a narrative and theoretical study drawing on psychoanalytic and sociological frameworks (Bion 1961; Douglas, 1992; Freud, 1913, 1921; Girard, 1986; Kirwan, 2005; Menzies Lyth, 1960; Reason, 2000; Zehr, 2015). It integrates case material selected for its prominence in public discourse and its illustrative value in revealing scapegoating dynamics. Interpretations were refined through peer discussion and theoretical reflection.

While this paper focusses particularly on the UK context, the dynamics of scapegoating of clinicians after tragedy are internationally widespread. It concludes that only by confronting the hidden rituals of modern sacrifice can we begin to build a just culture, one in which health professionals can care without fear, and where reparation is prioritised over blame, and learning over punishment.

## Scapegoating

. . . when individuals come together in a group all their individual inhibitions fall away and all the cruel, brutal and destructive instincts, which lie dormant in individuals as relics of a primitive epoch, are stirred up to find free gratification.

Freud (1921, p. 78)

Scapegoating has deep historical and psychological roots. Its symbolic origin lies in the biblical ritual from Leviticus, where a goat, burdened with the community’s sins, is cast into the wilderness. This act displaces unbearable emotion onto a single totemic figure to restore a fragile sense of order. Through this sacrifice, collective guilt is managed, and cohesion briefly restored.

Though scapegoating may offer short-term relief and emotional satisfaction, its effects are corrosive over time. It disrupts the group’s capacity to think clearly, reflect honestly, and process emotional pain (Girard, 1986; Reason, 2000). The disturbance it seeks to expel does not disappear, it returns, intensified by unconscious guilt, “the return of the repressed” (Freud, 1915, p. 154). At some level, the group knows what it has done, and the pain resurfaces, often in more fragmented and persecutory forms.

Many theorists argue that society is founded on an act of scapegoating. Christianity offers a powerful example: Jesus, burdened with the sins of the community, is executed. His resurrection exposes the violence as illegitimate and gives rise to a new moral order (Allport, 1949; Daniel, 1998; Freud, 1913; Girard, 1986; Volkan, 2004).

This foundational myth also reveals the enduring psychological appeal of scapegoating, not just as a cultural mechanism, but as a deeply rooted psychic response to suffering and moral confusion. Scapegoating is not only a defence but also reflects a deeper, unconscious pull towards retributive justice, the ancient logic of “an eye for an eye.” Though it offers a sense of moral balance, it serves vengeance more than truth (Zehr, 2015). It answers to the punitive superego, the harsh internal judge, evoked in the image of the Old Testament God who demands punishment for suffering (Freud, 1913, 1915). By contrast, restorative justice represents a more evolved moral stance: it asks us to face painful truths, acknowledge shared responsibility, and seek healing through dialogue (Zehr, 2015). However in moments of fear and loss, it is the more primitive instinct that takes hold. The urge to expel pain by punishing someone, anyone, remains deeply embedded in the collective psyche (Freud 1913, 1915).

## The Drive to Blame After Unpredictable Tragedy

I assumed the intention was to understand what had happened and not to find a fall guy.

Consultant psychiatrist after homicide (Hussain et al., 2024)

Healthcare is periodically confronted by rare and devastating events, a suicide, homicide, maternal death, or sudden catastrophic deterioration. However different their causes, they share a common psychic impact, exposing clinicians and society to the same unbearable reality: that not all deaths can be foreseen or controlled. Despite decades of research, for example, it remains impossible to predict who will go on to die by suicide or commit homicide (Gibbons, 2023, 2025a; Hawton & Pirkis, 2024; Large et al., 2016). These shocking events, arise unexpectedly and confront us with the disturbing unpredictability of existence. They shatter the assumptive belief that life is safe or orderly, leaving all involved in a state of profound shock and uncertainty about how and why the tragedy has occurred (Gibbons, 2024a; Parkes, 1988).

This psychic state is intolerable and within it the capacity for mentalisation (the ability to understand and interpret mental states) and symbolism is lost. It is defensively managed by constructing powerful, fixed narratives, *delusional narratives of blame* (Gibbons, 2024b), that alleviate pain and uncertainty. These blame narratives arise from an emotionally impaired, *non-mentalising* (Bateman, 2004) state of mind and are reductionist and linear, collapsing unbearable complexity into a false sense of comprehension and control. Oversimplifying the unpredictability of human experience, if we identify a cause, perhaps we can prevent recurrence. The “if only we had done X” story rests on speculation. It is emotionally compelling, but profoundly reductive. Healthcare offers no neat causal chains, no linear accounts that survive scrutiny. Every action is shaped by countless unknowns. Reducing tragedy to a single cause may ease anxiety, but it distorts truth, flattens complexity, and erodes compassion.

When grief can be tolerated, the psychological hold of these blame narratives loosens, allowing for a more nuanced understanding of agency and responsibility. The concept of *responsibility* belongs to a mentalising state of mind, one that recognises multiple, interacting causes and the limits of human control. The problem is that when these individualised narratives are reinforced by colleagues, organisations, media, and society, they harden, obstructing mourning, and fuelling broader dynamics of punishment and retribution (Gibbons, 2024a; Girard, 1986; Zehr, 2015).

## Scapegoating in Healthcare

We came to realize that the complaints stem from a collusive system of denial, splitting, and projection that is culturally acceptable to, indeed culturally required of, nurses. Each nurse tends to split off aspects of herself from her conscious personality and to project them into other nurses.

Menzies Lyth (1960)

Organisational scapegoating is not a rare event, but a recurring “system” of defence (Menzies Lyth 1960). It enables institutions, and those within them, to avoid confronting their own limitations and the unbearable realities of human nature and mortality, while simultaneously enforcing loyalty and conformity at times of institutional anxiety (Daniel, 1998; Dekker, 2023; Kirwan, 2005; Reason, 2000).

A sudden, serious incident triggers overwhelming anxiety and creates what Girard (1986) calls a *mimetic crisis* within the system, the fear that “this could have been me.” The anxiety generated quickly collapses complexity into a *linear narrative of blame*, a simple accusation of a missed form, an unchecked observation, a broken protocol. The event is no longer told with the confusion and fear it evokes but is recast as a story of individual failure (Kirwan, 2005). In this way, the contagion of anxiety described by Girard becomes codified through institutional language. Danger is never neutral but always moralised and modern systems of risk and accountability translate this anxiety into bureaucratic procedure (Douglas, 1992).

Scapegoating within organisations takes two forms: *expressive scapegoating*, a symbolic purge of group anxiety, and *instrumental scapegoating*, a strategic move by those in power to deflect scrutiny and resist change (Bonazzi, 1983; Cooke, 2007). In expressive scapegoating, the victim is blameless, a ritual sacrifice. In instrumental scapegoating, the target is marked by a perceived flaw around which blame coheres. They become a “rubbish bin” for unwanted projections such as, carelessness, neglect, and vulnerability. It becomes *them*, not me, not us (Klein, 1956, 2018). As Kirwan (2005) writes, “the victim becomes the embodiment of all evil and appears to the mob to be responsible for the crisis.” The scapegoat becomes both victim and supposed culprit, a container for systemic distress, offering emotional resolution while deflecting real insight or reform (Bonazzi, 1983; Freud, 1913; Girard, 1986).

An organisational investigation typically follows such events, but these processes are fundamentally flawed. Examining a complex system only after a tragic, equally complex outcome almost guarantees that problems will be found. Rarely is care assessed against a comparable pathway elsewhere. Instead, it is measured against the investigation team’s internalised fantasy of perfect care (Gibbons, 2025b). Healthcare is far from perfect. On some days, whether on wards, in emergency departments, or intensive care, multiple patients may be in acute crisis, each inhabiting and acting out their own inner world while simultaneously influencing, and being influenced by, others. Without working in these environments, it is difficult to grasp, just how chaotic, complex, and unpredictable they truly are (Blundy, 2011; Whittle, 2025).

In reality, the investigation holds two opposing tasks. The first is a healthy *work* developmental task (Bion, 1961), to use the energy of the trauma to examine the care pathway in good faith, learn from it, and support service improvement. This approach recognises that the causal factors leading to serious outcomes such as suicide, homicide, or unexpected death are, by nature, unknowable. The purpose of the investigation is not to speculate on these causes, as any conclusions drawn in this regard are understood to be hypothetical.

The second, unconscious, *basic assumption* task arises from the more primitive state of mind, (a regressive group state driven by shared anxiety and the need for emotional relief rather than truth) driven by the group’s need for certainty and emotional resolution (Bion, 1961; Gibbons, 2025b).

Here, the investigation becomes a vehicle for confirming a *delusional blame narrative* about what caused the tragic event (Gibbons, 2024b), to find fault, assign responsibility, and identify a scapegoat. The greater the anxiety, the more this unconscious task takes over.

Serious problems in health care, chronic under-resourcing and structural neglect, are undeniable (Gibbons, 2025b) however when blame shifts from individuals to an individual health organisation, the same dynamics of avoidance and oversimplification often persist.

In recent years, entire health organisations in the UK have become recurrent targets of public condemnation, cast as “bad organisations.” These include: Southern Health, Essex Partnership, Tees, Esk and Wear Valleys, Nottingham, Sussex Partnership, NELFT, the Priory Group, and Cygnet. Investigations have exposed various systemic and individual shortcomings including: falsified records, ignored investigations, poor risk assessments, missed ligature points, failed observations, and neglected supervision. However, many staff often privately admit that their own services and organisations are not so different and the same problems would be likely to be found under similar scrutiny. The problem is structural, not isolated, and scapegoating one organisation serves, as with individuals, to shield the wider system from confronting this truth.

Despite decades of awareness of its destructive effects, scapegoating persists because it fulfils powerful unconscious and organisational functions. At times of crisis, it channels collective anxiety into a manageable form, transforming diffuse guilt and helplessness into moral certainty (Menzies Lyth, 1960; Reason, 2000). For individuals, it restores mental balance; for leaders, it restores a sense of control; for institutions, it reaffirms the belief that order can be maintained if someone is held accountable; and for society, it offers the reassuring illusion that tragedy is preventable, that chaos can be contained, and that someone, somewhere, could have stopped it, if only they had acted differently.

## Who Is Selected as the Scapegoat?

To be marked was not a matter of innocence or guilt, but of being different from one’s fellows and unaware of that difference.

(Daniel, 1998, p. 22)

Scapegoat selection is rarely rational. It is shaped by unconscious dynamics, historical grievances, and identity-based projections (Girard, 1986; Volkan, 1985). In institutions, the most exposed are often the most vulnerable, those with the least power: nurses more than doctors, isolated staff, or already marginalised clinicians, particularly Black, Asian, and Minority Ethnic individuals (Williams, 2018). However, some scapegoats are not marginal figures at all, but highly reputed and valued practitioners, leaders in their field, esteemed by patients and colleagues, who become symbolic carriers of institutional anxiety and difference (Daniel, 1998). These scapegoated individuals are often placed in impossible roles, expected to maintain unachievably high standards and emotional control in chaotic, high-risk settings. These clinicians are caught in double binds, facing contradictory demands where any choice can later be framed as failure (Bonazzi, 1983; Cooke, 2007).

### Case Examples: High Profile Cases of Scapegoating in the UK, India, Australia, and USA

**UK: Dr Hadiza Bawa-Garba and nurse Isabel Amaro**

The 2011 convictions of Dr Hadiza Bawa-Garba and nurse Isabel Amaro for gross negligence manslaughter, following the death of six-year-old Jack Adcock, marked a turning point in the prosecution of healthcare professionals in the UK. Both clinicians had exemplary records and were working under extreme systemic pressure, Bawa-Garba during a 13-hour shift on her first shift after maternity leave, and Amaro on her 11th consecutive day as an agency nurse. While there were human errors, delayed administration of antibiotics, a brief misidentification during resuscitation, imperfect documentation and monitoring, expert reviews concluded that they were not the cause of death which was a result of sepsis, contributed to by systemic delays. Nonetheless, both clinicians were convicted and remain so (Bawa-Garba v GMC 2018; Hodson, 2020).

Why this case led to prosecution, when many similar incidents do not, remains unclear (NHS Resolution, 2022). The contrasting outcomes for Bawa-Garba, who was ultimately reinstated following a national outcry, and Amaro, who received little support, endured prolonged isolation and multiple suicide attempts (Telegraph, 2022), highlight troubling disparities in professional hierarchy, regulatory culture, and in whose careers are deemed expendable (Ameratunga et al., 2019; Cooke, 2007; Health and Care Professions Council, 2019; Jha, 2018; NMC Defence Barristers, 2019).

Widespread concern over the case led to the Williams Review (2018), which found that fear of prosecution was undermining openness, reflection, and learning in healthcare. It called for a “just culture” that distinguishes individual error from systemic failure.

**India: Dr Kafeel Khan and the Gorakhpur Oxygen Tragedy**

In August 2017, over 60 children died at the Baba Raghav Das Medical College Hospital in Gorakhpur, India, after the oxygen supply ran out due to unpaid bills. Public outrage was immediate, and political pressure to identify those responsible intensified. Initially praised for his efforts to secure emergency oxygen cylinders, paediatrician Dr Kafeel Khan was soon suspended, arrested, and accused of negligence and corruption. He spent eight months in jail and was publicly vilified by the state government and sections of the media. Yet 10 independent investigations and later court findings revealed that the deaths resulted from systemic failings, underfunding, administrative negligence, and lack of crisis preparedness, rather than individual error.

Dr Khan was eventually acquitted of the main charges, but only after his professional reputation had been destroyed and his family subjected to harassment (Dasgupta, 2022; Khan, 2021). His scapegoating performed an instrumental function restoring public confidence while shielding political and institutional culpability.

**Australia: Harry Bailey and Winifred Childs**

In Australia, the Chelmsford scandal became a defining moment for healthcare scapegoating. At its centre was Dr Harry Bailey, who developed and oversaw deep sleep therapy, a controversial treatment combining barbiturate-induced comas and electroconvulsive therapy. Between the 1960s and 1980s, at least 24 patients died at Chelmsford Private Hospital. Consent was often absent, records were falsified, and institutional oversight failed. Although many were complicit, Bailey became the instrumental scapegoat, the focal point of public and professional outrage and died by suicide in 1985, just before a Royal Commission began its inquiry. The Commission revealed widespread negligence and professional silence. Bailey became a symbolic sacrifice, absorbing the collective shame of a profession in crisis (Daniel, 1998)

In the same professional climate, Dr Winifred Childs, a respected and outspoken psychiatrist, was accused of forming an inappropriate emotional and sexual relationship with a former patient. The case against her relied largely on hearsay. Her public humiliation served a ritual function, restoring professional authority through the punishment of nonconformity (Daniel, 1998).

**USA: Dr. Anna Pou**

In the aftermath of Hurricane Katrina, Dr. Anna Pou remained at Memorial Medical Centre in New Orleans for four days, caring for dying patients amid extreme conditions, no power, 100°F heat, collapsing infrastructure, and no functioning emergency systems. When evacuation failed and death for some patients seemed inevitable, Dr. Pou administered morphine and sedatives to relieve suffering. After 45 bodies were recovered from the hospital, she and two nurses were arrested and charged with second-degree murder. Many viewed her prosecution as a clear act of scapegoating: by targeting Pou, the Louisiana Attorney General deflected public anger away from the underlying systemic failures, catastrophic government mismanagement, delayed federal response, inadequate disaster planning, and the abandonment of vulnerable populations. The case drew national attention and although a grand jury refused to indict her, the reputational and professional damage was done (Bailey, 2010).

## Scapegoating Disguised as “Accountability”

Words in healthcare are often used in inverted ways, as if their meanings are understood and shared, but under scrutiny, they frequently conceal more than they reveal. “*Risk*” becomes shorthand for suicide or homicide. “*Failings*” imply deviation from an imagined ideal of flawless care. “*Learning*” often means identifying and condemning imperfection rather than fostering reflection. “*Safety*” is invoked with conviction, yet rarely examined, how, for instance, can someone be kept safe from the contents of their own mind?

“*Accountability*” is another such word. We hear: “Someone must be held accountable,” but what do we mean? Who is accountable, for what, and how? When grief and fear are raw, calls for accountability can become euphemisms for punishment, a socially sanctioned demand for a scapegoat (Gibbons, 2024a).

## The Media’s Role in “Exciting” Primitive Scapegoating Dynamics

After tragic events, analysis of media language and focus reveals its role in encouraging primitive group dynamics, weaponising grief and transforming it into moral outrage. This discourse often unconsciously stigmatises mental illness, reduces the complexity of psychiatric care to a simplistic story of avoidable harm, and casts clinicians as murderers. To illustrate, consider two recent examples from the UK.

Benjamin Aninakwa, a ward manager, was convicted under the Health and Safety at Work Act following the death by suicide of Alice Figueiredo. A quote from her grieving mother, “Alice was effectively killed due to an appalling level of negligence,” was widely reported without context (Sky News, 2025). A similar pattern emerged in the case of Valdo Calocane, a man who had been under psychiatric care and who tragically murdered three members of the public. After a critical CQC report into the care provided, the BBC opened its segment with the line: “Blood on their hands. That is how the families . . . described the Trust’s failure.” Subsequent political responses, such as Wes Streeting’s call to name the individual psychiatrists involved in his care, further fuelled this primitive urge to identify a scapegoat (BBC Radio 4, 2024).

## The Bystander Effect, or Unconscious Collusion, in Scapegoating After Tragedy

Despite growing recognition that individuals and services are scapegoated after tragic outcomes, a collective paralysis often sets in. A silence descends, echoing the bystander effect, where witnesses fail to intervene due to diffused responsibility and fear of judgement (Darley & Latané, 1968).

Colleagues, institutions, and professional bodies often avoid challenging scapegoating narratives, fearing exposure to attack or retribution. There is a deep, often unspoken anxiety: that defending a clinician may appear lacking in compassion for bereaved families or undermine public trust in healthcare.

Beneath this lies a deeper belief, that retributive justice is not just expected, but necessary. Speaking out against scapegoating then becomes an act of whistleblowing, demanding courage to break institutional silence and risk isolation.

## Conclusion

The tumbrils begin to discharge their loads. The ministers of Sainte Guillotine are robed and ready. Crash!—A head is held up, and the knitting-women, who scarcely lifted their eyes to look at it a moment ago, count one. The second tumbril empties and moves on; the third comes up. Crash!—And the knitting-women, never faltering or pausing in their work, count two.

A Tale of Two Cities. Dickens (2003, p. 251)

This paper illustrates that scapegoating functions as a primitive defence against unbearable psychic pain, displacing collective anxiety onto vulnerable individuals, obstructing learning, undermining the credibility of legal and regulatory systems, and perpetuating systemic dysfunction. More disturbingly, it exposes our vulnerability to primitive impulses, when faced with grief and distress, we do not seek understanding, we seek sacrifice. We look for scapegoats.

To unconsciously perpetuate the scapegoating of clinicians for events that are, by nature, complex, multifactorial, and beyond control is to endanger the future of health care. Failing to confront this dynamic risks a culture in which few will be willing to practise, and fewer still prepared to care for those in their most desperate moments. Who will step forward in an open hearted manner when caring risks professional ruin, public condemnation, or criminal prosecution? Continuing to allow colleagues to face manslaughter charges and imprisonment after unpredictable tragedy, means accepting that any one of us, or those we work with, could be next.

A common challenge is whether focussing on scapegoating lets dangerous individuals “bad apples”, or dysfunctional systems “bad orchards” escape scrutiny (quote from Deshpande, 2025). But scapegoating doesn’t illuminate, it obscures. It diverts us from genuine responsibility, disguising stigma as analysis. It replaces inquiry with blame, making it harder, not easier, to identify real systemic or individual problems. Negligence and poor care exist and must be addressed. But when a simplistic causal narrative is imposed on a complex event, leaving one individual exposed, it must be challenged.

What is lost in the drive for blame is not just the depth of the work, but its emotional, moral, and existential complexity. In clinging to the fantasy that tragic events are preventable if only the right action had been taken, we deny a painful truth: some outcomes are beyond our control.

Our professional and public cultures remain steeped in the hidden rituals of modern sacrifice. The demand for retribution may feel justified to the primitive epoch residing within us all, but it is not justice, it is an act of psychic violence (Girard, 1986). Health services are asked to contain what is, in truth, uncontainable: the destructiveness and unpredictability of human nature itself (Gibbons, 2025b). The hardest, most honest stance is to say: we do not know, and to bear that not-knowing without retreating into blame or control. Death, tragedy, and human destructiveness are real, and scapegoating a clinician does nothing to change it.

## Implications for Policy and Practice

Following a serious event, if an individual is rapidly named or blamed, it must be assumed that scapegoating is taking place, until proven otherwise. This should become the default stance for clinical services, investigations, and regulators. The questions outlined in Box 1 can help identify when scapegoating dynamics are unfolding: Who is being singled out? What systemic conditions are being ignored? Why this person, in this way, at this time?

**Box 1.** Questions to Ask When Someone Is Identified After a Serious Event

1. Did the accusation emerge after a tragic and fundamentally unpredictable event? ● Was this a tragic event that no one could have reliably predicted? ● Did the response begin before space for reflection or containment was possible? *→ Scapegoating often follows shocking events where uncertainty and distress are intolerable.*
2. Is a simplified, linear narrative being constructed? ● Has the complexity of the event been reduced to a single cause or error? ● Does the story imply that one person, or one missed action, could have prevented the outcome? ● Has the care been compared with an idealised version of perfect care, rather than with a comparable service or standard? *→ Scapegoating depends on overly neat stories that obscure complexity and unpredictability.*
3. Was the narrative formed in the immediate aftermath, during emotional upheaval? ● Is the emotional tone charged with urgency, outrage, or moral certainty? ● Has grief been acknowledged, or is it being displaced through blame? *→ High emotional intensity and rushed narrative closure are warning signs of scapegoating.*
4. Has blame replaced thought? ● Are terms like “fault” and “blame” dominating the discussion? ● Is there pressure to suspend, discipline, or remove someone before full understanding has been reached? *→ Scapegoating bypasses thinking in favour of emotional discharge and symbolic resolution.*
5. Is the focus exclusively on the individual, rather than the system? ● What broader context shaped the person’s role—staffing, supervision, organisational culture? ● Have others involved been overlooked? Why? *→ Scapegoating isolates; genuine accountability requires systemic reflection.*
6. Is the individual vulnerable or unsupported? ● Are they junior, marginalised, or working alone? ● Were they assigned an impossible task with inadequate support or oversight? *→ Scapegoats are often those least able to defend themselves.*
7 Who benefits from the blame? ● What emotional relief or institutional stability does the blaming provide? ● What systemic issues are being avoided, denied, or deflected? ● Has further inquiry or reform been halted or redirected? *→ Scapegoating may offer short-term relief, but undermines long-term trust, learning, and change.*

Scapegoating must be recognised as a serious organisational risk and routinely inquired about at board level. This allows the destructive dynamics operating beneath the surface to be brought into conscious awareness, where they can be thought about, named, and worked with, rather than simply acted out through disciplinary procedures. Left unexamined, scapegoating undermines staff morale, obstructs learning, and harms rather than helps those bereaved after tragedy.

Leaders, investigators, and policymakers must develop the reflective capacity to recognise its signs and actively resist its pull. Only by fostering environments that can tolerate uncertainty, complexity, and emotional pain can we begin to move from rituals of punishment to cultures of inquiry, repair, and shared responsibility.

## Limitations and Future Research

As an interpretive and theoretical study, this paper inevitably reflects the author’s clinical and organisational perspective. The analysis is based on secondary case material and public documentation rather than primary data, and its purpose is conceptual rather than empirical validation. Future empirical work could test these propositions by examining how scapegoating dynamics are experienced and narrated within contemporary healthcare organisations, using qualitative and ethnographic methods to explore how blame, responsibility, and institutional defence are enacted in practice.
